# Correlation between Ultrasound Elastography and Histologic Characteristics of Papillary Thyroid Carcinoma

**DOI:** 10.1038/srep45042

**Published:** 2017-03-22

**Authors:** Li Yi, Wu Qiong, Wang Yan, Fan Youben, Hu Bing

**Affiliations:** 1 Department of Ultrasound in Medicine, Shanghai Institute of Ultrasound in Medicine, Shanghai Jiao Tong University Affiliated Sixth People’s Hospital, Shanghai, China; 2 Department of General Surgery, Shanghai Jiao Tong University Affiliated Sixth People’s Hospital, Shanghai, China

## Abstract

The aim of this study was to investigate the correlation between elastography and histologic characteristics including fibrosis and calcification. We also wanted to investigate whether other clinicopathologic indexes influence the strain ratio (SR) of papillary thyroid carcinomas (PTCs). We retrospectively reviewed 126 papillary thyroid carcinomas (PTCs) from 103 patients who underwent ultrasonography and elastography before surgery. The histologic characteristics and clinicopathologic indexes were compared with the SR of ultrasound elastography (UE). The results showed that there was a significantly positive correlation between fibrosis degree and SR measurements (r = 0.754, p = 0.000); the SR was significantly different between the groups with and without calcification (11.34 ± 10.08 vs. 6.81 ± 7.33, p = 0.000). The standard coefficients of collagen and stromal calcification were 0.684 and 0.194, respectively. There was no significant correlation between SR and indices such as size, position, co-existence with Hashimoto’s thyroiditis (HT), multifocality or cervical lymph node (CLN) metastasis. In conclusion, we found that the SR of UE is positively correlated with the fibrosis of PTC. Stromal calcification will elevate the SR dramatically, but psammoma bodies will not when they exist in the absence of stromal calcification.

The diagnosis of thyroid cancer has increased rapidly, with a 240% increased incidence over the past three decades^1^. Papillary thyroid carcinoma is the most common type of thyroid cancer and is the most common endocrine malignancy, accounting for 96.0% of all total new endocrine cancers and 66.8% of deaths due to endocrine cancers^1^,^2^. The vast majority of thyroid cancers present as thyroid nodules that are incidentally detected in an ultrasonography imaging exam of the neck for other disorders. High-resolution ultrasonography is considered the most effective exam method for detecting and diagnosing thyroid cancer. Elasticity is one of the most important physical properties of human tissue, and assessing the elasticity could reveal more information to facilitate diagnosis.

Ultrasound elastography (UE) has been introduced as a method to evaluate tissue elasticity^3^, and the good diagnostic performance of UE has been gradually accepted by radiologists and clinicians^4^,^5^,^6^. The latest American Thyroid Association (ATA) guidelines mentioned UE as a promising technique^7^. The Strain Ratio (SR) offers semi-quantitative information about the stiffness of thyroid nodules, which is obtained by the reference tissue strain divided by the lesion strain^8^. A higher SR indicates a harder tissue. UE is also known as “electronic palpation” because it can predict papillary thyroid carcinoma (PTC) from benign nodules^5^.

The best cut-off for SR in diagnosing papillary thyroid carcinoma varies from 0.78 to 3.28 according to a variety of different studies; considering our own experiences, we determined the optimal cut-off for SR to be >2.58 9. Further, we found that the SR of one type of thyroid cancer, PTC, can vary from 2.0 to more than 50.0. However, we still do not know why SR could vary so much. We are not sure if this variance is due to unreliable measurements, differential histologic characteristics, or different backgrounds or positions of the nodule. It is also well known that the elasticity of the nodules is closely associated with extracellular matrix (ECM) components, particularly the collagens and some cytokines that can promote collagen expression and tissue fibrosis^10^. Therefore, the huge discrepancy seems to be explained by differential collagen content. According to our own experience, we also hypothesized that calcification could be an additional reason for the observed variance.

Therefore, in this study, we intended to investigate the correlation between elastography and histologic characteristics such as fibrosis and calcification. We also wanted to investigate whether other clinicopathologic indexes such as size, position, co-existence with Hashimoto’s thyroiditis (HT), or the presence of multiple foci or cervical lymph node (CLN) metastasis potentially influence the SR in PTCs.

## Results

There were 75 female subjects and 28 male subjects in our study. The mean age of the 103 subjects was 35.87 ± 11.14 years (range, 24–67 years). The average SR of the PTCs was 8.82 ± 8.91 (range: 1.6~53.4). HT was confirmed in 20.39% (21/103) of the patients, and 24.27% had positive diagnoses in the central/later part of the neck lymph nodes. The general patient profile is shown in Table 1.

**Figure 1:**
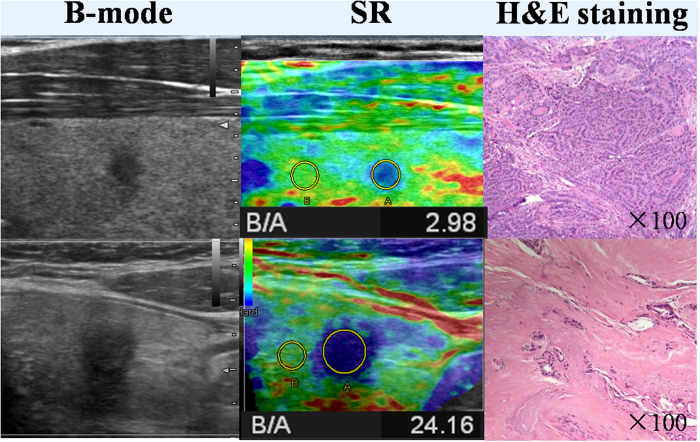
The first row shows the B-mode ultrasonography and histologic image of one PTC. SR of the PTC is 2.98 and the H&E staining slide shows the nodule is cell dominant type. The second row shows the B-mode ultrasonography and histologic image of another PTC. SR of the PTC is 24.16 and the H&E staining slide shows the nodule is collagen dominant type.

**Figure 2:**
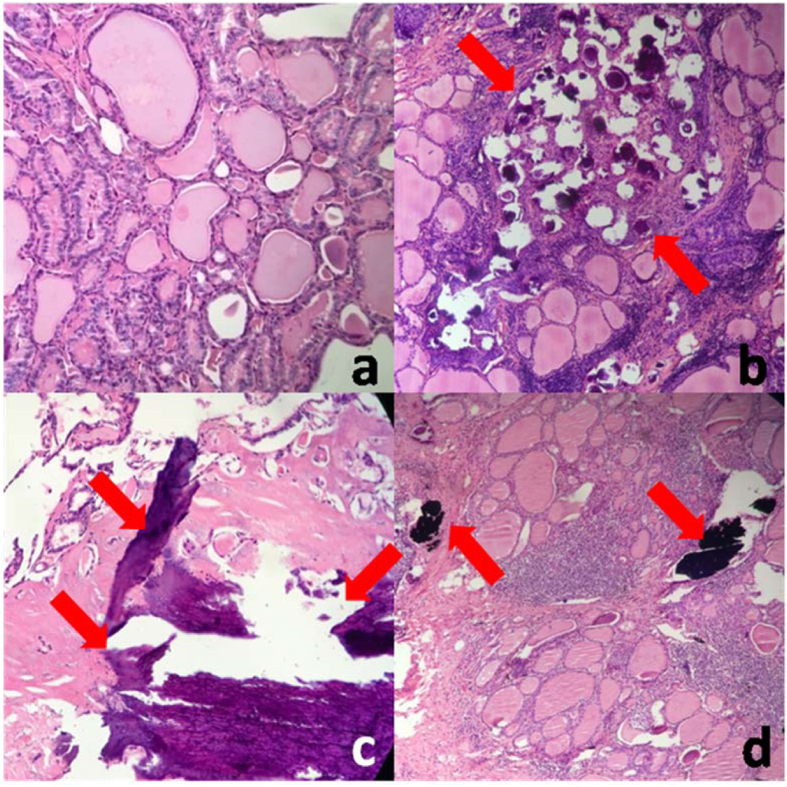
Different types of calcification. (**a**) PTC without calcification. (**b**) PTC with only psammoma bodies. (**c**) PTC with only stromal calcification. d. PTC with both psammoma bodies and stromal calcification. Red arrows show the positon of calcification.

**Figure 3:**
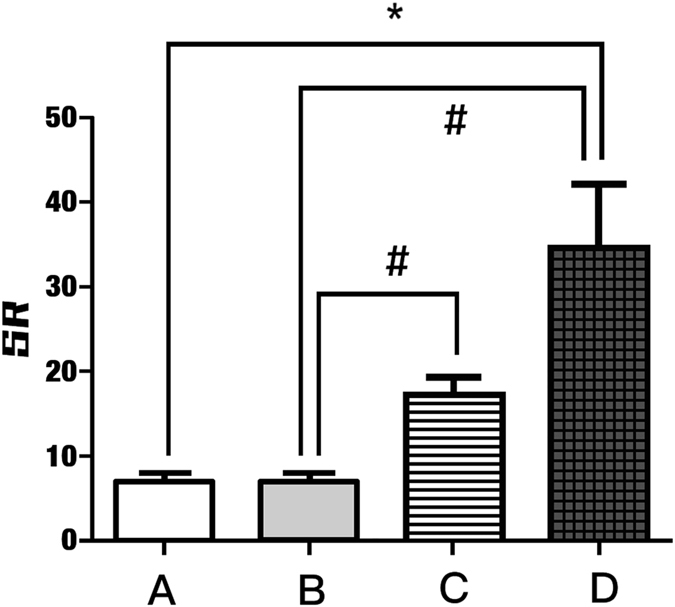
SR in PTCs with different types of calcification. Group A: nodules without calcification; Group B: nodules only with psammoma bodies; Group C: nodules only with stromal calcifications; Group D: nodules with both psammoma bodies and stromal calcifications (Fig. 2). *SR of Group D was significantly higher than Group A. ^#^Group D and Group C were significantly higher than Group B.

1. The correlation between elastography and histologic characters in PTC

1. The correlation between elastography and fibrosis in papillary thyroid carcinoma. There was a significantly positive correlation between fibrosis degree and SR measurements (r = 0.754, p = 0.000, Fig. 1).
2. The correlation between elastography and calcification in papillary thyroid carcinoma. The calcification could be classified as three types: psammoma bodies, stromal calcifications or bone formation, as noted in the methods. In our study, the bone formation type was absent. Among the 56 nodules with calcification, only the psammoma body and stromal calcification types were observed. The SR was significantly different between the groups with and without calcification (11.34 ± 10.08 vs. 6.81 ± 7.33, p = 0.000). In the 56 nodules with calcification, there were 35 nodules with psammoma bodies only, 6 nodules with stromal calcifications only and 15 nodules with both types of calcification. All of the nodules were classified into 4 groups according to the histologic findings: Group A: nodules without calcification; Group B: nodules with psammoma bodies only; Group C: nodules with stromal calcifications only; and Group D: nodules with both psammoma bodies and stromal calcifications (Fig. 2). The SR of Group D was significantly higher than those of the other 3 groups (27.02 ± 15.51 vs. 7.28 ± 3.50 and 6.81 ± 7.33; p = 0.012, 0.000, respectively). The SR of Group C was significantly higher than those of Groups A and B (17.22 ± 4.22 vs. 7.28 ± 3.50 and 6.81 ± 7.33; p = 0.012, 0.023, respectively). However, there was no significant difference between Groups C and D (27.02 ± 15.51 vs. 17.22 ± 4.22, p = 0.092). In addition, there was no significant difference between Groups A and B (7.28 ± 3.50 vs. 6.81 ± 7.33; p = 0.999; Tables 2 and 3, Fig. 3).
3. The influence of fibrosis and calcification on elastography in PTC

According to multivariate regression analysis, collagen and stromal calcifications all had significantly positive influence on the SR of PTC. The standard coefficients of collagen and stromal calcification were 0.684 and 0.194, respectively. The influence of collagen was stronger than that of calcification in PTC when elastography was performed.

2. Analysis of other possible clinicopathologic influencing factors of elastography in papillary thyroid carcinoma

We evaluated the correlation between SR and the characteristics of papillary thyroid carcinoma, such as size, position, multifocality and co-existence with HT or lymph node metastasis. According to the chi-square test, we did not find a significant correlation between the SR and the indices mentioned above (Table 4).

## Discussion

Stiffness is a key property of abnormal tissues and organs. Previous *ex vivo* and *in vivo* studies have shown that malignant thyroid tumors have a 10-fold greater stiffness than normal tissues do^8^. Therefore, a firm and hard thyroid nodule upon palpation is known to be associated with an increasing risk of thyroid malignancy^11^. Previous studies have shown that the stroma component is the most important factor that contributes to nodule stiffness. In PTC, obvious fibrosis could be observed in 50~70% of the nodules, and some of the fibrotic nodules were accompanied by calcification or bone formation. Therefore, we supposed that fibrosis and calcification would affect the elasticity of nodules and attempted to prove this hypothesis. In our study, we confirmed that the collagens in the stroma are key to the elastography results. The more collagen there is in the stroma, the higher the SR will be.

It is not difficult to understand that in malignant tumors, invasive tumor cell behavior causes more complicated stromal reactions, and the stromal reaction will result in collagen remodeling (converting normal flexible fiber into high-risk stiff aligned collagen, which could make the nodules “hard”)^12^. Compared with malignant tumors, the thyroid adenomas and nodular goiter were much softer and expressed much less collagen^13^. Most benign nodules grow slowly and have no invasive behavior; therefore, the stromal reaction is much weaker, and the nodules are softer. Another reason that should not be neglected is that papillary thyroid carcinoma can contain many papillae in which there are abundant collagen axes. The unique structure of the papillary thyroid could also increase the stiffness of PTCs in addition to stromal fibrosis.

It is not surprising that calcification is also a very strong influential factor in the UE results, particularly stromal calcification. Among PTCs with different types of calcification, the SR measurements were much higher when stromal calcification was present. However, psammoma bodies seem have little influence on the elasticity. The underlying observation is that calcification, especially macro-calcification including stromal calcification, can appear in benign thyroid nodules such as follicular adenomas and nodular goiters and is occasionally seen in anaplastic thyroid carcinoma. Therefore, it is very important for doctors to know that benign nodules can also get a higher “score” in UE. When performing UE, avoiding the area of calcification is very important.

Therefore, we have determined the correlation between pathologic characteristics and UE. We can easily understand why the false positive and false negative UE results appear and how to better interpret the UE assessments. If the tumor grows too fast and causes partial liquidation, the general stiffness will decrease, except for the solid portions of the papillae. Thus, it should be very discreet to choose Area A. Some types of PTC lack the typical papillae structure; therefore, the stiffness may decrease to such an extent that the follicular variant PTC, which will cause a false negative in UE^7^,^14^. Meanwhile, it is not difficult to understand the false UE positives. Because the nodule stiffness is closely related to fibrosis and stromal calcification, some adenomas and nodular goiters with calcification could present as hard nodules, which may result in a false positive UE assessment.

According to our study, co-existence with HT seems have no effect on the SR result. One of our studies showed that HT will cause fibrosis of the thyroid, which would decrease the SR because the SR measurement is obtained by dividing the reference tissue strain by the lesion strain. However, this decrease would not influence the SR enough to change the existing diagnostic standard or cut-off SR value for PTCs^9^. The results described here were also consistent with those of other studies^15^. We also tried to investigate whether there was a correlation between lymph node metastasis and SR, but the result was still unclear. In our study, 44.44% (12/27) of the PTCs with positive lymph nodes had an SR less than 5, whereas 33.33% (33/99) of the PTCs without positive lymph nodes had SRs less than 5. It seems that the softer nodules were more likely to demonstrate positive lymph nodes. This could indicate that softer PTCs are inclined to be more aggressive. This finding is not coherent with the findings obtained for breast cancer by Andrew Evens, in which elasticity was positively correlated with lymph node metastasis according to a linear model. However, in the multiple linear regress analysis, they found that only invasive size was the stronger factor that would greatly influence the stiffness of the cancer.

The limitation of this study was that we did not use a more specific staining method to evaluate the collagen content. The positive area proportion in the H&E stained slides could miss some thin collagens, and Masson staining might be a better choice to evaluate the collagen proportions.

In conclusion, we found that the SR of UE is positively correlated with PTC fibrosis. Stromal calcification will elevate the SR dramatically, but psammoma bodies will not when they exist without stromal calcification. Size, position, co-existence with HT, multifocality and positive cervical lymph nodes have no significant relationship to SR.

## Material and Method

### Patients

We retrospectively reviewed the patients who underwent total/partial thyroidectomy in our hospital from January 2013 to December 2015. The inclusion criteria were those patients who were histologically confirmed as a classic type of PTC and had undergone ultrasonography and elastography before surgery in our hospital. All of the ultrasonography and elastography procedures were performed by one Radiologist (Dr. YW). In her clinic, elastography is a routine exam for every patient with thyroid nodules. The exclusion criteria were (1) nodules without reliable elastograms (nodules with coarse calcification or on the position of isthmus); (2) rare types of PTC; (3) nodules without SR ratios. Finally, we included 126 nodules from 103 patients. For each patient, the central lymph nodes were dissected during surgery, and the dissection of the lateral neck lymph nodes was used as an alternative depending on the ultrasound findings. Informed consent was obtained from all patients, and this study was approved by the Ethics Committee of the Shanghai 6th People’s Hospital.

### Imaging

We retrospectively reviewed all of the elastograms and elastography reports written after the exams. All UE exams were performed by one doctor (Dr. YW) who had more than 25 years of thyroid US scanning experience and at least 4 years of experience performing real time elastography on solid thyroid lesions. We confirmed that all methods were performed in accordance with the relevant guidelines and regulations^16^,^17^,^18^.

#### Protocol of conventional ultrasound and UE

For each patient, bilateral thyroid lobe sonography was performed in the transverse and longitudinal planes using a Hitachi HV-900 or Avius (Hitachi Medical, Tokyo, Japan) US scanner equipped with a sonoelastography unit and a 7.5~13.0 MHz linear-array transducer. In the B-mode US, she first examined the target lesion and stored the images of the lesion. Several B-mode US features such as position (in one of the lobes or in the isthmus; in the center of the gland or near the capsule), echogenicity (hyper-, iso- or hypo-), margin (clear or vague), shape (A/T > 1 or < 1) and types of calcification (micro- or coarse) were recorded (micro-calcification was defined as calcification less than 1 mm)^19^. The elastograms and strain ratio measurements were performed immediately after conventional US using the same real-time instrument and the same probe with a freehand technique. The patients were asked to remain relaxed and keep still. The real-time elastograms were acquired at 15 frames per second. The best frame was indicated by a green compression bar, and the optimal frame was then saved and analyzed. An area A (covering the target lesion that had sufficient surrounding area of normal thyroid tissue) was selected and then, in the same frame, an area B (surrounding area, at the same depth and same size as long as possible) was also selected. The SR measurement is obtained by the reference tissue strain divided by lesion strain, and it can be automatically calculated by the ultrasound system immediately after the doctor finishes choosing the target lesion and reference lesion. Then, the SR measurement will be displayed on the screen. Each lesion was assessed at least three times, and the average SR measurement was recorded and written on a report. All of the images were stored on the hardware. To ensure objectivity, the mean SR measurements were recorded without knowing the pathological results. The central lymph nodes were dissected during the surgery, and the dissection of the lateral neck lymph nodes was completed depending on the ultrasound findings. In this study, we also collected the results of the thyroid function test before the surgery.

### Histological characteristics

The diagnosis of PTC was based on histological samples of surgical resection. Lymph node metastasis was also confirmed by histological findings. The diagnosis of HT was based on lab results, imaging appearance and histological findings. On the H&E stained slides, the collagen appeared as apparent fibrous stroma bands among cancerous cells. At a 200x high magnification field, we chosen 3 random views, and using the ImageJ software (Rawak Software, Inc. Germany), we calculated the percentage of collagen in one view and then calculated the average ratio based on 3 views per 200x field. The calcifications were classified as three types: psammoma bodies, stromal calcifications and bone formation. The presence of psammoma bodies was defined as spherical calcified foci with concentric laminations^20^,^21^,^22^, and these were usually located within stromal stalks of tumor papillae distinct from the intrafollicular inspissated colloid^20^,^21^. Bone formation was regarded as positive only when both the bone matrix and osteocytes were identifiable. All calcific masses that did not meet the criteria of psammoma bodies or bone formation were categorized as stromal calcifications. Blank cancerous cells were also indirect evidence of the presence of stromal calcification because some of these cells could be washed out during the slicing and paraffin procedure.

### Statistical analysis

All statistical analyses were calculated using the Statistical Package for Social Sciences (SPSS for Windows, version 19; Chicago, IL, USA). Means and S.D. were used to summarize quantitative variables, whereas frequencies and percentages were used to summarize qualitative data. The relationships between independent variables (size, position, calcification, single or multifocal, combined with HT or not, degree of fibrosis and lymph nodes status) and SR measurements were studied using a general linear model (Fisher’s exact analysis was used when the frequency was less than 5). Multiple linear regression analysis was used to perform multivariate analysis to determine which pathologic variables were most influential on mean stiffness readings. A Pearson test and a correlation coefficient were calculated to evaluate the relationship between fibrosis degree and SR. The ANOVA test was performed to compare quantitative data from more than 2 groups. A correlation coefficient was also calculated. If the coefficient value was ≥ 0.7, coherence was considered strong; if the coefficient value was 0.4 ≤ value <0.7, coherence was considered moderate; and if the value was <0.4, coherence was considered weak. P < 0.05 was considered to indicate a significant difference.

## Additional Information

**How to cite this article:** Yi, L. *et al*. Correlation between Ultrasound Elastography and Histologic Characteristics of Papillary Thyroid Carcinoma. *Sci. Rep.* **7**, 45042; doi: 10.1038/srep45042 (2017).

**Publisher's note:** Springer Nature remains neutral with regard to jurisdictional claims in published maps and institutional affiliations.

## Figures and Tables

**Table 1:** Demographics of the Study Population of PTC.

Category		
Sex	Male	28
	Female	75
Age (years old)		35.87 ± 11.14 (range: 24–67)
Surgery on thyroid before	Yes	4
	No	99
Thyroid hormones level	Normal	86
	Abnormal	17
Thyroglobulin antibodies	Normal	87
	Elevated	18
Thyroid peroxidase antibody	Normal	82
	Elevated	21
Taking levothyroxine	Yes	7
	No	96

**Table 2:** The different SR of elastography between PTC with and without calcification.

Calcification	No	Yes
Ratio of subjects	55.56% (70/126)	44.44% (56/126)
SR	6.81 ± 7.33	11.34 ± 10.08^*^
P value	0.000	

*Significantly higher than the group without calcification.

**Table 3:** The different SR of elastography among PTC with differen types of calcification.

Group	A	B	C	D
Calcification	No	Only with psammoma bodies	Only with stromal calcifications	With both psammoma bodies and stromal calcifications
Ratio of subjects	55.56% (70/126)	27.78% (35/126)	4.76% (6/126)	11.90% (15/126)
SR	6.81 ± 7.33	7.28 ± 3.50	17.22 ± 4.22*^,#^	27.02 ± 15.51*^,#^

*Significantly higher than group A. ^#^Significantly higher than group B.

**Table 4:** Relationships between SR and Clinicopathologic Features 126 PTCs.

Reference parameter	SR ≤ 5 (45)	5 < S ≤ 10 (52)	S > 10 (29)	t value	P value
Size					
≤1 cm (57)	18	28	11	2.679	0.262
>1 cm (69)	27	24	18		
In isthmus					
Yes (7)	5	2	0	4.643	0.098
No (119)	40	50	29		
Positon					
Upper lobe (6)	3	1	2	6.236	0.134
Middle lobe (114)	42	48	24		
Buttom lobe (6)	0	3	3		
Position					
Near front capsule (14)	2	11	1	8.153	0.477
middle (96)	37	35	24		
Near back capsule (16)	6	6	4		
Multi-focal					
Yes (12)	4	6	2	0.459	0.861
No(114)	41	46	27		
Co- existin with HT					
Yes (21)	6	11	4	1.191	0.560
No (105)	39	41	25		
Cervical lymph nodes metastasis					
Yes (27)	12	11	4	1.671	0.449
No (99)	33	41	25		
